# Dr. Samuel A. Cartwright

**Published:** 1852-10

**Authors:** 


### DR. SAMUEL A. CARTWRIGHT.

Dr. Cartwright is one of the most eminent physicians of the South. A great many years ago he published, in the Medical Recorder, papers on Bilious Fever, which won for him a national reputation. He is an independent, original thinker, a bold, clear, vigorous writer, and a practitioner of remarkable solidity of judgment. He is greatly esteemed for the excellence of his moral character, his probity, simplicity, and amiability in private life, not less than for his professional learning and skill. We have been much interested in some controversies in which he has lately become involved, by his advocacy of certain novel views in anatomy and physiology. First, he performed an operation upon a female laboring under disease of the ovarium, in which he believed he had succeeded in passing a catheter along one of the Fallopian tubes into that organ. Dr. Brickell questioned the reality of the performance, and this has brought on an angry discussion. We are sorry that the discussion has assumed this character. There can be no question that both gentlemen are conscientious. Dr. Cartwright seriously believes that he did catheterize the Fallopian tube; there is no doubt in the world of that. Dr. Brickell does not believe that such an operation is practicable, but in expressing a doubt on this point it was not his intention to call in question the veracity of Dr. Cartwright. It is a matter about which doubts are very naturally entertained; but Dr. Cartwright may safely leave his reputation for veracity to take care of itself. No one will doubt that if he made a mistake about this operation, it was an honest mistake.

Our friend has warmly embraced Mrs. Willard's theory of the circulation of the blood, and this has involved him in a controversy with another party. He displays great tact, gallantry, spirit, and address, in his support of the hypothesis of this "*Filia Nata Jovis*," as he styles the fair authoress, and though he may have the worst of the argument, he renews the contest with an alacrity worthy of a veteran as he is. One cannot but admire the courage with which he returns to the contest after he had seemed to have been overwhelmed by his adversary. But, we must say, there appears to us but small hope for the doctor and his "*Hematokinety*," with such antagonists as Dr. Ely, Dr. Riddell, and above all, Dr. Dowler, "the alligator king." This discussion, however, will go on, as all scientific discussions should, good-naturedly. The readers of the New Orleans medical Journal have had a treat in the articles which it has called forth. Dr. Cartwright's last is full of humor and vivacity.

Last, but not least, Dr. Cartwright has "a philosophy of the negro constitution," which has called out a swarm of critics. He is a true believer in the inferiority of the negro race. He holds to slavery as a Bible doctrine. He insists that the Africans, as the sons of Ham, are doomed to bondage, and that it is a violation of the highest law to attempt to set them free. They are inferior, he maintains, not only from habit and moral influences, but from a law of organization which can neither be reversed nor amended. The sum of his doctrine is that they have less mind because they consume less oxygen; that they are not only not capable of breathing as much air, but do not incline to breathe as pure air, and hence, when they sleep, "cover their heads with a blanket." These views—this "philosophy of the negro constitution"—he defends stoutly and resolutely against assailants of whatever creed, and from whatever quarter. He is in earnest. He believes every word of the doctrine, and in defence of it, evinces no common ingenuity and research, and most uncommon resolution. Numbers do not intimidate him, nor is he awed by authorities however high or venerable. We apprehend, however, that his philosophy is not likely to gain much favor. It is assailed in the very quarter where it seemed most probable that it would meet with advocates—in the extreme South—and with a force of argument and fact which our learned friend will find it difficult to resist.

We like Dr. Cartwright's independence, and his industry is to be commended. We are sure that we express the feelings of all his brethren, not excepting his critics, when we wish that he may live yet as many years as he has already spent in honorable professional labor.

